# Soy Isoflavone Protects Myocardial Ischemia/Reperfusion Injury through Increasing Endothelial Nitric Oxide Synthase and Decreasing Oxidative Stress in Ovariectomized Rats

**DOI:** 10.1155/2016/5057405

**Published:** 2016-02-01

**Authors:** Yan Tang, Shuangyue Li, Ping Zhang, Jinbiao Zhu, Guoliang Meng, Liping Xie, Ying Yu, Yong Ji, Yi Han

**Affiliations:** ^1^Key Laboratory of Cardiovascular Disease and Molecular Intervention, Nanjing Medical University, Nanjing 210029, China; ^2^Department of Gynaecology, The First Public Hospital of Zhangjiagang, Zhangjiagang 215699, China; ^3^Aoyang Hospital, Zhangjiagang 215600, China; ^4^Department of Pharmacology, School of Pharmacy, Nantong University, Nantong 226001, China; ^5^Key Laboratory of Food Safety Research, Institute for Nutritional Sciences, Shanghai Institutes for Biological Sciences, Chinese Academy of Sciences, Shanghai 200031, China; ^6^Department of Geriatrics, First Affiliated Hospital of Nanjing Medical University, Nanjing 210029, China

## Abstract

There is a special role for estrogens in preventing and curing cardiovascular disease in women. Soy isoflavone (SI), a soy-derived phytoestrogen, has similar chemical structure to endogenous estrogen-estradiol. We investigate to elucidate the protective mechanism of SI on myocardial ischemia/reperfusion (MI/R) injury. Female SD rats underwent bilateral ovariectomy. One week later, rats were randomly divided into several groups, sham ovariectomy (control group), ovariectomy with MI/R, or ovariectomy with sham MI/R. Other ovariectomy rats were given different doses of SI or 17*β*-estradiol (E_2_). Four weeks later, they were exposed to 30 minutes of left coronary artery occlusion followed by 6 or 24 hours of reperfusion. SI administration significantly reduced myocardial infarct size and improved left ventricle function and restored endothelium-dependent relaxation function of thoracic aortas after MI/R in ovariectomized rats. SI also decreased serum creatine kinase and lactate dehydrogenase activity, reduced plasma malonaldehyde, and attenuated oxidative stress in the myocardium. Meanwhile, SI increased phosphatidylinositol 3 kinase (PI3K)/Akt/endothelial nitric oxide synthase (eNOS) signal pathway. SI failed to decrease infarct size of hearts with I/R in ovariectomized rats if PI3K was inhibited. Overall, these results indicated that SI protects myocardial ischemia/reperfusion injury in ovariectomized rats through increasing PI3K/Akt/eNOS signal pathway and decreasing oxidative stress.

## 1. Introduction

Several researches have demonstrated that there are gender differences in the risk of cardiovascular diseases, such as premenopausal women exhibiting a lower risk than age-matched men, but it disappears after menopause [1–3]. It suggests a special role for estrogens in preventing and curing cardiovascular disease in women. As we all know, estrogen replacement therapy (ERT) can attenuate the incidence of cardiovascular events in postmenopausal women [4]. However, several large-scale, randomized, controlled studies have questioned the beneficial effects of ERT in postmenopausal women in many epidemiologic studies [5]. The conflicts have stimulated further research on alternative treatments, such as phytoestrogens. Soy isoflavone (SI), a soy-derived phytoestrogen, is a group of biologically active plant substances with chemical structures which are similar to that of an endogenous estrogen-estradiol [6]. The similarity of structures might partly explain why these compounds have the ability of binding to estrogen receptors (ERs) and exerting various effects.

As the development of thrombolytic therapy or percutaneous coronary intervention in clinic, the incidence of myocardial ischemia/reperfusion (MI/R) injury increases. As well as we know, MI/R is an important complication of acute arterial occlusion and subsequent recanalization. Moreover, in the presence of clinical condition, recanalization is used as an attempt to minimize the infarct area; however, the reoxygenation of ischemic heart usually leads to functional loss on the area of myocardial [7–9]. So researchers paid more and more attention to reduce I/R injury. Recently, it is also gradually appreciated that there were distinctions of different gender on sensitivity and/or severity in ischemia/reperfusion (I/R) induced injuries in heart, liver, kidney, and so on [10, 11]. With consistent findings, females are less susceptible to I/R induced injuries compared with males. 17*β*-estradiol was reported to protect against myocardial I/R injury in female Wistar rats [12]. Meanwhile, some reports indicated estrogen binding to ER lessened the extent of injury during MI/R after ovariectomy [13]. Previous studies suggested that estrogen receptor mediated protective effect on female MI/R injury was associated with phosphatidylinositol 3-kinase (PI3K)/protein kinase B (Akt)/endothelial nitric oxide synthase (eNOS) signaling cascade [14, 15].

On the other hand, reactive oxygen species (ROS), as highly reactive compounds causing peroxidation of lipids and proteins, play an important role in the pathogenesis of MI/R injury [16, 17]. ROS have been long-term recognized to enhance oxidative stress and activate I*κ*B (inhibitor of NF-*κ*B)/p-I*κ*B/nuclear factor-*κ*B (NF-*κ*B) pathway, which was considered as a classic signaling pathway. NF-*κ*B remains in an inactive state in the cytoplasm in normal station because of a complexing to its inhibitor-I*κ*B. As response to stress factors, for example, I/R, different signaling pathways converge on the activation of I*κ*B kinase complex (IKK), which induces phosphorylation of the I*κ*B. Paralleling with the loss of I*κ*B in the cytoplasm is the appearance of NF-*κ*B in the nucleus [18].

Therefore, the purpose of the present study was to determine whether SI protects myocardium from I/R injury through activating PI3K/Akt/eNOS and reducing oxidative stress pathway. If so, a new ideal would be provided for postmenopausal I/R injury and even other cardiovascular diseases in clinic.

## 2. Materials and Methods

### 2.1. Animals and Experimental Groups

Animal experiments were conducted according to the Guidelines of Committee for the Care and Use of Laboratory Animals of Nanjing Medical University. Female Sprague Dawley (SD) rats (180~220 g) were obtained from Beijing Vital River Laboratory Animal Technology, Co, Ltd (Beijing, China). Rats were anesthetized with chloral hydrate (3.5 mL/kg, intraperitoneal injection) and underwent bilateral ovariectomy (OVX) as previously described [19]. The sham procedure of ovariectomy consisted of administration of anesthesia and visualization of the ovaries through incisions into the abdominal cavity and closure of the wounds.

One week after ovariectomy, the rats were randomly divided into eight groups: sham ovariectomy operation (control group), ovariectomy with sham MI/R (OVS group), or ovariectomy with MI/R (OVX group). Other ovariectomy rats were given different doses of soy isoflavone (SI) dissolved in 0.5% carboxymethylcellulose (CMC-Na) by gavage (L-SI group: 30 mg/kg·d, M-SI group: 90 mg/kg·d, H-SI group: 270 mg/kg·d). Additional ovariectomy rats were administrated with the same volume of CMC-Na by gavage (CMC group) or 50 *μ*g/kg·d of 17*β*-estradiol (E_2_) by subcutaneous injection (OVE group) instead of drug once daily over the same period. All rats fed soy-free chow (corn starch: 32%, sucrose: 30%, casein: 23%, corn oil: 5%, cellulose: 5%, mixing mineral salts: 3.5%, mixing vitamin: 1%, DL-methionine 0.3%, and choline dipl-tartrate: 0.2%) and water ad libitum. To block the PI3K pathway in vivo, an intraperitoneal injection of a PI3K inhibitor 2-(4-morpholinyl)-8-phenyl-1(4H)-benzopyran-4-one hydrochloride (LY294002, Sigma-Aldrich, MO, USA, diluted in CMC for injection) at dose of 0.3 mg/kg was given immediately after SI administration.

### 2.2. Surgical Procedures of MI/R

**Figure 1:**
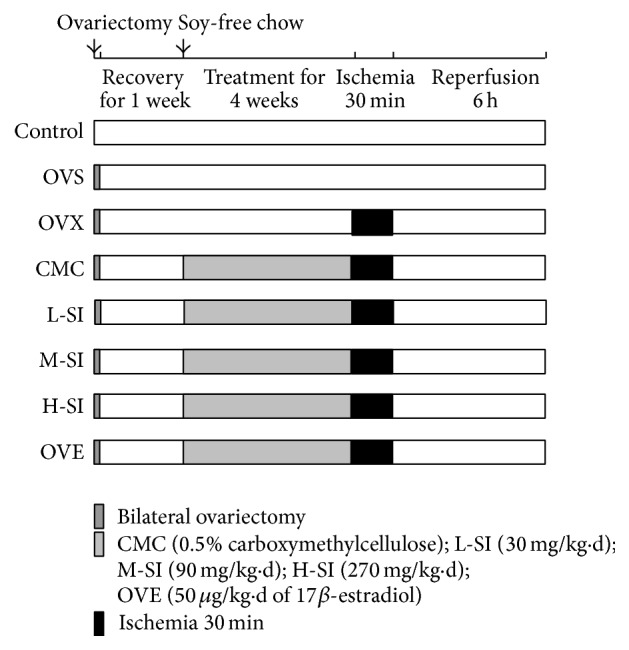
Experimental procedure. One week after ovariectomy, rats were maintained with soy free chow and given different treatments. Four weeks later, rats were subjected to 30 min myocardial ischemia and 6 h or 24 h reperfusion.

After 4-week treatment, MI/R was performed as described previously [20] (Figure 1). All rats except control group were anesthetized with sodium pentobarbital (40 mg/kg, intraperitoneal injection). Then, atropine of 0.1 mg/kg was administered to reduce airway secretions by subcutaneous injection. Intraoperative monitoring of adequate anesthesia was done by toe pinching. A core body temperature of 37°C was maintained by automatic heating blanket during surgery. After exposing heart, myocardial ischemia (MI) was produced by temporarily exteriorizing the heart via a left thoracic incision and placing a 6-0 silk suture with a slipknot around the left anterior descending coronary artery. After 30 minutes of MI, the slipknot was released and the myocardium was reperfused for 6 hours (only for cardiac function analysis) or 24 hours (for infarct size determination and other measurements). A sham operation was subjected to the same surgical interventions without performing occlusions (OVS group). Blood and tissue samples were obtained after reperfusion for future analysis.

### 2.3. Echocardiography

After 6 hours of reperfusion, cardiac function was evaluated using an echocardiography system (Visual Sonics Vevo 2100, VisualSonics, CA) equipped with a 12-MHz linear-array transducer. Two-dimensional (2D) images were obtained in the parasternal long axis view. Left ventricular (LV) ejection fraction (EF) and fractional shortening (FS) were derived by goal-directed, diagnostically driven software. All measurements were made by one observer who was blinded to the identity of the treatments. The values were averaged over five consecutive cardiac cycles.

### 2.4. Determination of Myocardial Infarct Size

Myocardial infarct size was determined by Evans blue/triphenyltetrazolium chloride (TTC) staining. Briefly, the hearts were removed and perfused with saline on a Langendorff system to wash blood from the coronary vasculature, followed by staining with 1.5% Evans blue to determine the area at risk. The heart was sliced horizontally into five slices with similar thickness. The slices were incubated in 1.2% TTC prepared with Tris Buffered Saline (TBS, pH 7.8) for 15 min at 37°C. Then, viable nonischemic myocardium stained blue with Evans blue staining, while ischemic but viable myocardium stained red with TTC and necrotic myocardium stained pale white.The infarct area (white) and the area at risk (red plus white) from each section were determined using an Alpha EaseFC image analyzer (Alpha Innotech Corporation. CA, USA). Ratios of risk area to left ventricle (RA/LV) and infarct area to risk area (IA/RA) were calculated as percentages.

### 2.5. Vascular Relaxation Responses

The thoracic aorta from rat was dissected out and carefully removed from adhering adipose and connective tissues. Vessels were then cut into approximately 4 mm ring segments with special care to reserve the endothelium and mounted into an organ bath (DMT, A/S Inc., Denmark) filled with Krebs solution (composition in mmol/L: NaCl 119, KCl 4.7, KH_2_PO_4_ 1.18, MgSO_4_ 1.17, NaHCO_3_ 25, CaCl_2_ 2.5, EDTA 0.026, and glucose 5.5, pH 7.4) at 37°C with 95% O_2_ and 5% CO_2_. The rings were attached to a force transducer and a resting tension of 9.8 mN was maintained throughout the experiment. For endothelium-dependent relaxation responses, vessel rings were preconstricted with noradrenaline (NE, Jinrao amino acid company, Tianjin, China) of 10^−7 ^mol/L. After the plateau was attained, the rings were exposed to different concentrations of acetylcholine (ACh, 10^−9 ^mol/L to 10^−5 ^mol/L, Sigma, USA) to obtain cumulative concentration-response curves.

### 2.6. Measurement of Serum Creatine Kinase (CK), Lactate Dehydrogenase (LDH), Estradiol, and Plasma Malondialdehyde (MDA)

Blood samples were collected from the right carotid artery. After centrifuging at 1000 g for 15 min, the supernatant was obtained for various assays. Serum CK and LDH as well as plasma MDA levels were assessed according to the manufacturer's instructions by using commercially available kits (Jiancheng Biochemistry Co. Ltd., Nanjing, China). Serum estradiol levels were estimated by radioimmunoassay kit (Beijing North Institute of Biological Technology). Each measurement was performed in duplicate.

### 2.7. Western Blotting

Briefly, left ventricle of cardiac tissue was homogenized with a homogenizer in TBS buffer and then centrifuged at 8000 bpm for 5 min at 4°C. Precipitation was homogenized in tissue lysis buffer (NaCl 150 mmol/L; TRIS 25 mmol/L; NaF 50 mmol/L; Na_3_VO_4_ 1.0 mmol/L; phenylmethylsulfonyl fluoride 1.0 mg/L; aprotinin 1.0 mg/L; leupeptin 10 mg/L; pH 7.6) and then placed on ice for 50 min. After centrifugation at 12,000 bpm for 10 min at 4°C, the supernatants were removed and stored at −80°C. Protein concentrations were determined by the BCA method (Pierce Chemical, USA).

Prestained protein marker (New England Biolabs Ltd, Ontario, Canada) and 60 *μ*g protein samples were separated by 8% or 10% SDS-PAGE. The separated protein was transferred onto a 0.45 *μ*M polyvinylidene fluoride membrane (Millipore, USA). After blocking at room temperature in TBS containing 0.2% Tween 20 (TBS-T) and 5% nonfat dry milk for 2 h, the membrane was incubated with P85*α* (rabbit monoclonal, 1 : 1000, Cell Signaling Technology Inc., MA, USA), anti-phosphorylation-Akt (Ser473) (rabbit polyclonal, 1 : 2000, Cell Signaling Technology Inc. MA, USA), anti-Akt (rabbit polyclonal, 1 : 1000, Cell Signaling Technology Inc., MA, USA), anti-phosphorylation -eNOS (Ser1177) (rabbit polyclonal, 1 : 5000, Cell Signaling Technology Inc., MA, USA), anti-eNOS (mouse polyclonal, 1 : 2000, BD Biotechnology Inc., USA), anti-iNOS (rabbit polyclonal, 1 : 5000, Bioworld Technology Inc., MD, USA), anti-I*κ*B*α* (rabbit polyclonal, 1 : 5000, Stanza Cruz Biotechnology Inc., CA, USA), *β*-actin (mouse monoclonal, 1 : 4000, Sigma-Aldrich, MO, USA), or GAPDH (mouse monoclonal, 1 : 6000, Kang Cheng, China) in blocking buffer at 4°C overnight. Membranes were then washed 3 times in TBS-T buffer, followed by incubation with 1 : 6000 dilutions of horseradish-peroxidase-conjugated anti-rabbit IgG or 1 : 4000 dilutions of horseradish-peroxidase-conjugated goat anti-mouse IgG (Santa Cruz Biotechnology, CA, USA) at room temperature for 2 h and washing 3 times in TBS-T. Visualization was carried out using an Enhanced Chemiluminescence (ECL) kit (Thermo Fisher Scientific Inc., IL, USA).

### 2.8. Measurement of Superoxide Anion Formation

Hearts or 4 mm vessel rings of thoracic aorta removed from rats were immediately frozen in Tissue-Tek OCT embedding medium (Sakura Finetek, Tokyo, Japan). Then, the samples were cut into 5-*μ*m-thick sections and placed on glass slides. Dihydroethidium (DHE, 2 *μ*M, Beyotime, China), topically applied to each tissue section, was used to evaluate superoxide levels in situ. After the slides were incubated in a dark chamber at 37°C for 30 min, DHE fluorescences were observed by fluorescence microscope (Olympus, Japan).

### 2.9. Statistical Analysis

All data are expressed as mean ± SEM deviation and were analyzed by 1-way ANOVA followed by the Bonferroni* post host* test as appropriate (Stata13.0 software). Values of *P* < 0.05 were considered statistically significant.

## 3. Results

### 3.1. Effect of SI on Uterus Weight, Body Weight, and Serum Levels of Estradiol in Ovariectomized Rats

After ovariectomy, body weight increased significantly, which was consistent to the fact that higher body weight was in postmenopausal women [21]. Rats with SI (90 mg/kg·d and 270 mg/kg·d) or E_2_ (50 *μ*g/kg·d) administration show significant reduction in body weight. Uterine weight, used as a bioassay for estrogens [22], was measured after 4-week treatment (Table 1). Uterus of lighter weight and lower concentration of E_2_ were present after ovariectomy, which suggested that ovariectomy elicited estradiol deficiency and uterine atrophy (Table 1). After 4-week treatment, SI (270 mg/kg·d, H-SI group) and 17*β*-estradiol (50 *μ*g/kg·d, OVE group) administration increased estradiol level and uterus weight compared with CMC group (Table 1).

### 3.2. SI Improved Cardiac Function of Hearts with I/R in Ovariectomized Rats

**Figure 2:**
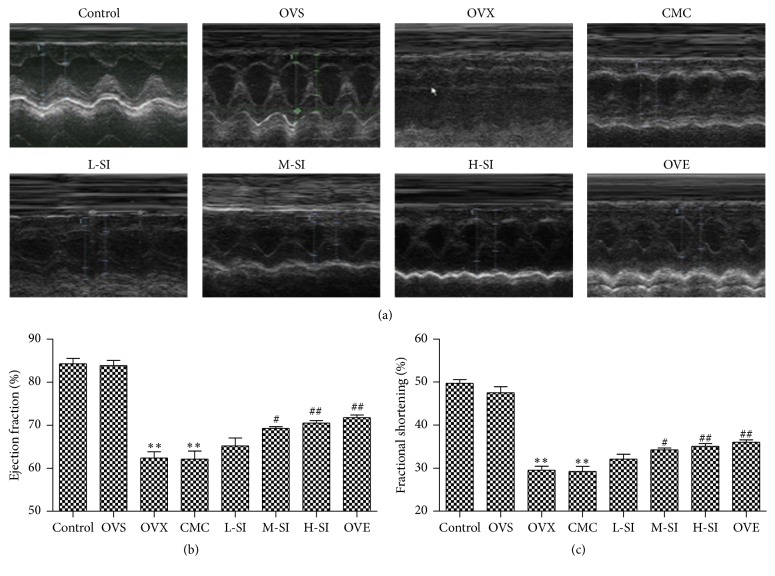
SI improved cardiac function after I/R in ovariectomized rats. (a) Representative echocardiography in each group after 6 h perfusion. (b) Ejection fraction after 6 h perfusion. (c) Fractional shortening after 6 h perfusion. Plots represent the mean ± SEM, *n* = 4–6. Statistical significance: ^*∗∗*^ *P* < 0.01 compared with OVS; ^#^ *P* < 0.05, ^##^ *P* < 0.01 compared with CMC.

According to the representative 2D echocardiographs after 6 h perfusion (Figure 2(a)), ejection fraction (EF) and fractional shortening (FS) decreased in OVX and CMC group compared with OVS group. There was an improvement in EF and FS in M-SI and H-SI group compared with CMC group, which reached the level in OVE group (Figures 2(b) and 2(c)).

### 3.3. SI Decreased the Infarct Size of Hearts with I/R in Ovariectomized Rats

**Figure 3:**
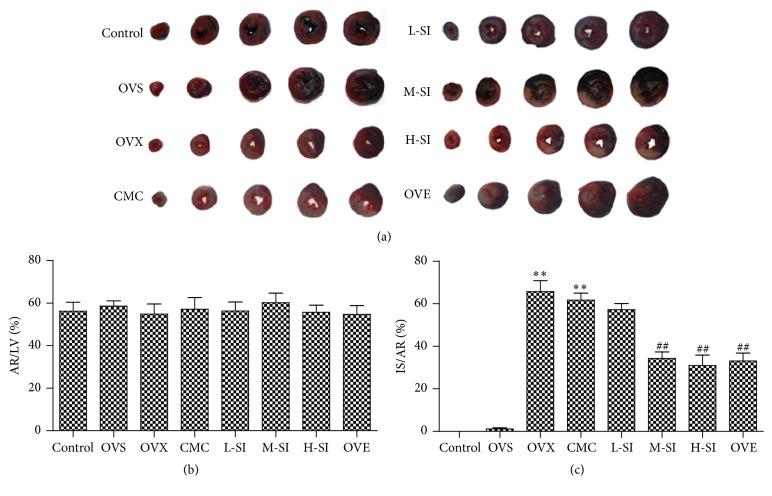
SI decreased MI size after I/R in ovariectomized rats. (a) Representative staining of heart by Evans blue/triphenyltetrazolium chloride (TTC) after 24 h perfusion. Evans blue-stained areas (blue) indicate nonischemic/reperfused area; TTC stained areas (red staining) indicate ischemic but viable tissue; Evans blue/TTC staining negative areas (white staining) indicate infarcted myocardium; red staining plus white staining indicates area at risk (AR). (b) Ratio of area at risk (AR)/left ventricle (LV) in each group after 24 h perfusion. (c) Ratio of area of infarction size (IS)/AR after 24 h perfusion. Plots represent the mean ± SEM, *n* = 3–5. Statistical significance: ^*∗∗*^ *P* < 0.01, compared with OVS; ^##^ *P* < 0.01 compared with CMC.

Compared with OVS group, myocardial infarction size was greater in OVX group, suggesting that 30 min ischemia followed by 24 h reperfusion resulted in significant myocardium infarct. The infarction size (IS)/AR was significantly smaller in M-SI group and H-SI group than that in CMC group, which was similar to the level of OVE group, suggesting that SI plays a protective role in the heat during I/R in ovariectomized rats (Figure 3).

### 3.4. SI Decreased Serum CK and LDH after I/R in Ovariectomized Rats

**Figure 4:**
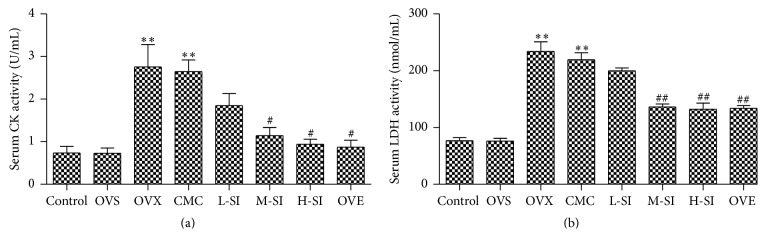
SI decreased serum CK and LDH activity after I/R in ovariectomized rats. (a) Level of serum CK activity. (b) Level of serum LDH activity after 24 h perfusion. Plots represent the mean ± SEM, *n* = 4–6. Statistical significance: ^*∗∗*^ *P* < 0.01 compared with OVS; ^#^ *P* < 0.05, ^##^ *P* < 0.01 compared with CMC.

Serum CK and LDH activity was evaluated to assess the extent of myocardial injury after I/R [23, 24]. A significant increase in CK and LDH activity was observed in OVX and CMC group compared with the OVS group. After 4-week treatment, there were lower levels of CK and MDA in M-SI and H-SI group compared with the CMC-treated rats, which reached the level in OVE group (Figure 4).

### 3.5. SI Upregulated Myocardial PI3K/Akt/eNOS Pathway during I/R in Ovariectomized Rats

**Figure 5:**
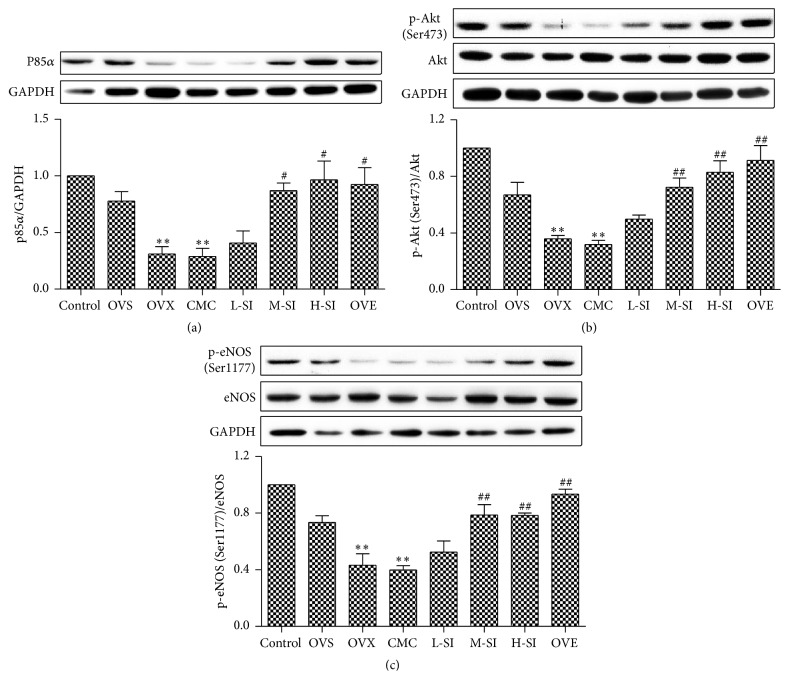
SI increased myocardial PI3K/Akt/eNOS pathway during I/R in ovariectomized rats. Expression of p85*α* (a), phosphorylation of Akt (Ser473) (b), and eNOS (Ser1177) (c) with western blotting after 24 h perfusion. Plots represent the mean ± SEM, *n* = 5-6. Statistical significance: ^*∗∗*^ *P* < 0.01, compared with OVS; ^#^ *P* < 0.05, ^##^ *P* < 0.01 compared with CMC.

To elucidate the protective mechanism of SI on myocardial I/R, expressions of PI3K/Akt/eNOS signaling molecules were determined in myocardium. The ovariectomized rats subjected to I/R demonstrated a significant decrease in protein expression of p85*α*, phosphorylation of Akt (Ser473), and eNOS (Ser1177) compared with OVS group. After 4-week treatment, there were great increases of above protein expressions in M-SI and H-SI groups compared with the CMC-treated rats, which reached the level in OVE group (Figure 5).

### 3.6. SI Attenuated Oxidative Stress in Myocardium after I/R Injury in Ovariectomized Rats

**Figure 6:**
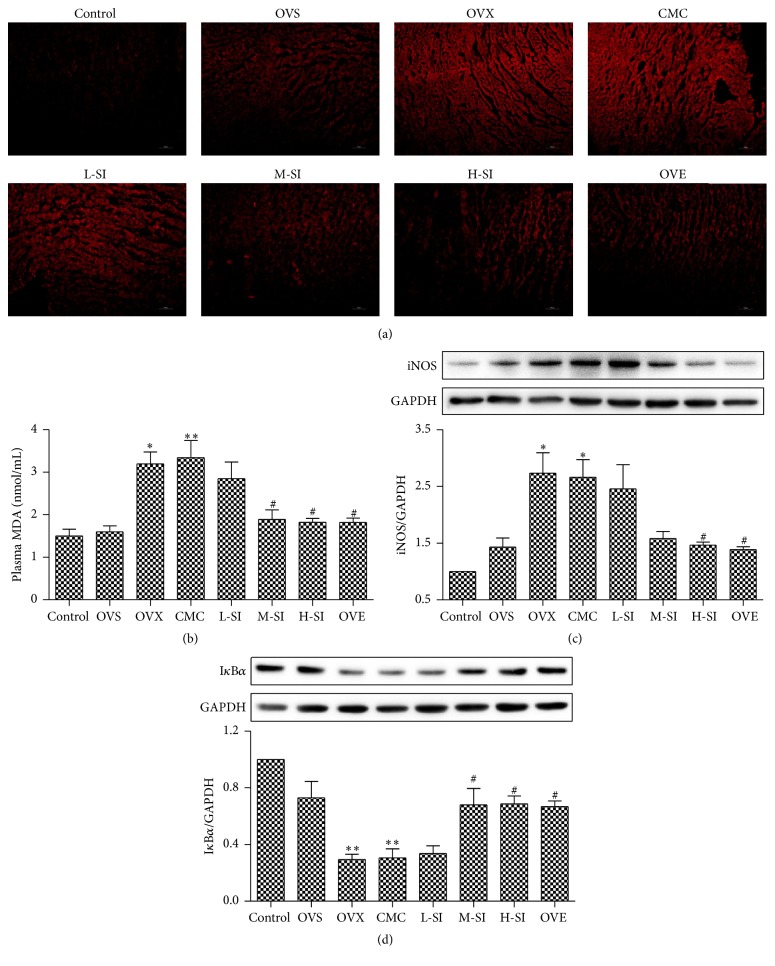
SI decreased myocardial oxidative stress and iNOS expression but increased I*κ*B*α* expression after I/R injury in ovariectomized rats. (a) Dihydroethidium (DHE) fluorescence staining of myocardium. (b) Lever of plasma MDA. ((c) and (d)) Expression of iNOS (c) and I*κ*B*α* (d) in myocardium with western blotting after 24 h perfusion. Plots represent the mean ± SEM, *n* = 6. Statistical significance: ^*∗*^ *P* < 0.05, ^*∗∗*^ *P* < 0.01 compared with OVS; ^#^ *P* < 0.05 compared with CMC.

Level of ROS reflected by the intensity of DHE fluorescence was much higher in the myocardium of OVX group compared with OVS groups. Four-week treatment with SI significantly reduced ROS production in the myocardium compared with the CMC-treated rats, which was similar to that in OVE group (Figure 6(a)). There was also a significant increase in plasma MDA in OVX and CMC group compared with the OVS group. After 4-week treatment, there were lower levels of MDA in M-SI and H-SI groups compared with CMC group, which reached the level in OVE group (Figure 6(b)).

### 3.7. SI Inhibited iNOS but Enhanced I*κ*B*α* Expression in Myocardium after I/R Injury in Ovariectomized Rats

There was higher expression of iNOS in OVX group compared with OVS group, and SI treatment significantly decreased it (Figure 6(c)). Meanwhile, the protein of I*κ*B*α* displayed opposite changes. There was lower expression of I*κ*B*α* in OVX group compared with OVS group, and SI treatment significantly enhanced it, which reached the level in OVE group (Figure 6(d)). All of these results suggested that SI attenuated myocardial oxidative damage after myocardial I/R injury in ovariectomized rats.

### 3.8. SI Improved the Endothelium-Dependent Relaxation of Thoracic Aortas in Ovariectomized Rats

**Figure 7:**
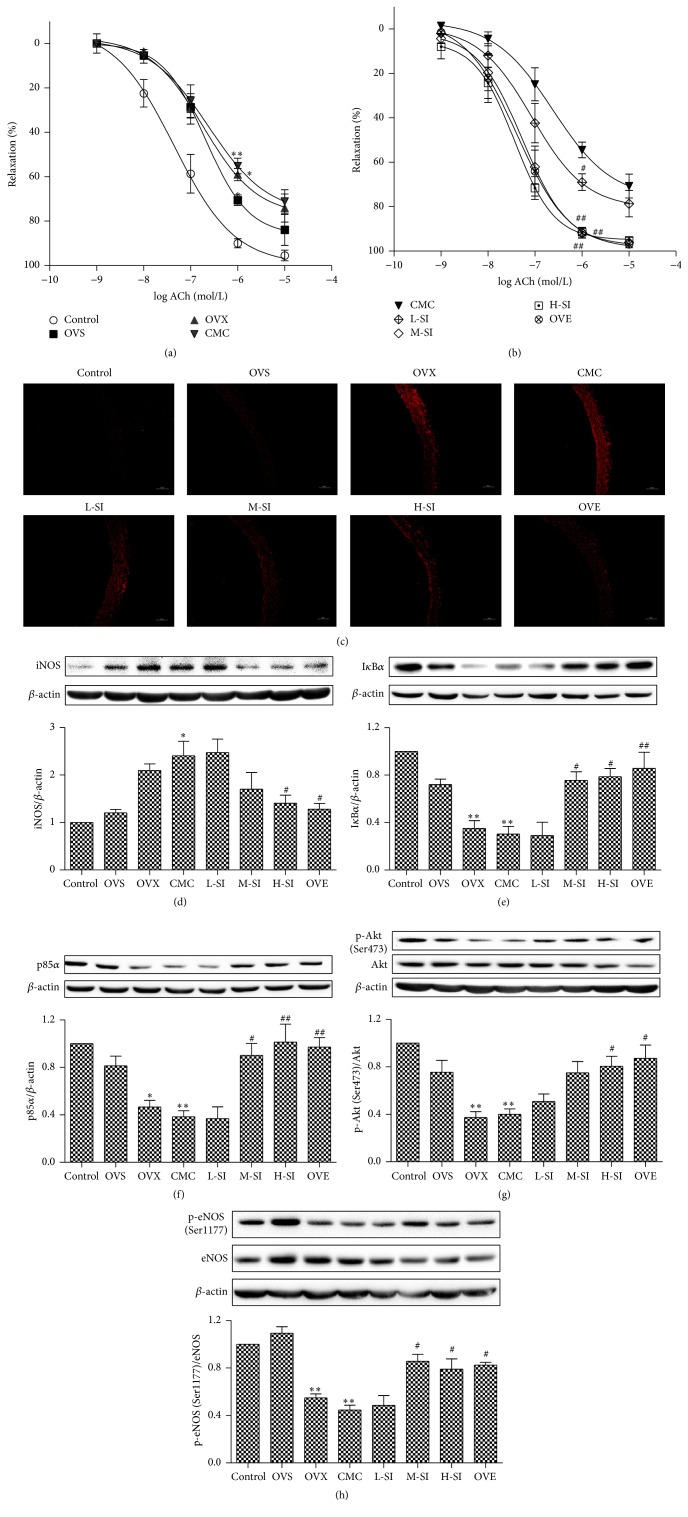
SI improved endothelium-dependent vasorelaxation, decreased oxidative stress and iNOS expression, and increased I*κ*B*α* expression and PI3K/Akt/eNOS phosphyration in thoracic aorta in ovariectomized rats. (a and b) Endothelium-dependent vasorelaxation to acetylcholine of precontracted aortic sections was assessed. (c) Dihydroethidium (DHE) fluorescence staining of thoracic aorta. ((d)–(h)) Expression of iNOS (d), I*κ*B*α* (e), and p85*α* (f), phosphorylation of Akt (Ser473) (g) and eNOS (Ser1177) (h) in thoracic aorta with western blotting after 24 h perfusion. Plots represent the mean ± SEM, *n* = 5–7. Statistical significance: ^*∗*^ *P* < 0.05, ^*∗∗*^ *P* < 0.01 compared with OVS; ^#^ *P* < 0.05, ^##^ *P* < 0.01 compared with CMC.

Endothelium-dependent relaxation of thoracic aortas to acetylcholine was remarkably impaired in ovariectomized rats after I/R injury compared with OVS group (Figure 7(a)). After four-week treatment, SI treated rats showed significant improvement of endothelium-dependent relaxation of thoracic aorta, which was similar to the effect of estradiol in ovariectomized rats (Figure 7(b)).

### 3.9. SI Attenuated Oxidative Stress in Thoracic Aortas after I/R Injury in Ovariectomized Rats

Level of ROS reflected by the intensity of DHE fluorescence was much higher in thoracic aortas of OVX group compared with OVS groups. Four-week treatment with SI significantly reduced ROS production in thoracic aortas, which was similar to that in OVE group (Figure 7(c)).

### 3.10. SI Suppressed iNOS but Elevated I*κ*B*α* and PI3K/Akt/eNOS Expression in Thoracic Aortas after I/R Injury in Ovariectomized Rats

After treatment, SI markedly decreased iNOS expression but increased expression of I*κ*B*α* and p85*α* and phosphorylation of Akt and eNOS in thoracic aortas (Figures 7(d)–7(h)). Combined with the above results, it was indicated that the improved function of thoracic aortas by SI may be related with inhibition of oxidative stress and improvement of PI3K/Akt/eNOS signaling pathway.

### 3.11. SI Failed to Decrease the Infarct Size of Hearts with I/R in Ovariectomized Rats If PI3K Was Inhibited

**Figure 8:**
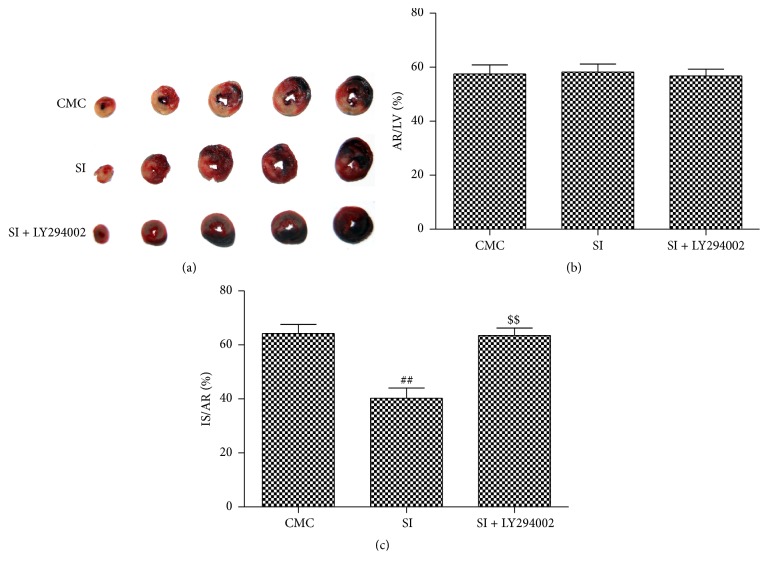
SI failed to decrease the infarct size of hearts with I/R in ovariectomized rats if PI3K was inhibited. One week after ovariectomy, the female SD rats were given 0.5% CMC (CMC group) or SI of 270 mg/kg·d by gavage (SI group). LY294002 (a specific PI3K inhibitor) at dose of 0.3 mg/kg was intraperitoneally injected immediately after SI was given (SI + LY294002 group). (a) Representative staining of heart by Evans blue/TTC after 24 h perfusion. (b) Ratio of area at risk (AR)/left ventricle (LV) in each group after 24 h perfusion. (c) Ratio of area of infarction size (IS)/AR after 24 h perfusion. Plots represent the mean ± SEM, *n* = 4. Statistical significance: ^##^ *P* < 0.01, compared with CMC; ^$$^ *P* < 0.01 compared with SI.

LY294002, a specific inhibitor of the PI3K/Akt pathway, was used to further demonstrate whether PI3K/Akt/eNOS pathway was essential in the attenuating effect of infarct size with I/R in ovariectomized rats. It is worth noting that SI during I/R in ovariectomized rats was not able to reduce the infarction size if PI3K inhibitor was administrated (Figure 8).

## 4. Discussion

As well as we know, after menopause, cardiovascular risk among women becomes progressive because of decreased levels of estrogens [25, 26]. Estrogen actions are essentially mediated by binding to estrogen receptors [27]. Isoflavone, with similar structure to estrogen, also acts on estrogen receptor to influence the cardiovascular system [28]. Myocardial I/R injury occurs following partial or complete cessation of blood circulation to the myocardium. Since Jennings et al. first described the phenomenon of MI/R injury in 1960 [29], previous researches have been trended to illuminate the mechanisms of MI/R injury and to investigate interventions on cardioprotection [30]. Despite the fact that increasing knowledge has been studied on the protection of MI/R, none of the experimental interventions has proved to be effective in the clinic [31]. All of these highlight the fact that MI/R injury is a complex pathological condition. An effective clinical therapy for MI/R remains a great challenge.

Several studies have showed that there were apparent gender differences of risk of cardiovascular diseases between men and women [32–34]. This difference might be attributed to the loss of female sex steroid hormones after menopause, but the biological explanations for gender differences in cardiovascular diseases, including myocardial I/R injury, are more complex. In our study, serum estradiol concentration and uterine weight decreased but body weight increased in ovariectomized rats, which imitate the menopause situation in women. Recent studies indicate that sex steroid hormones and their receptors are critical determinants of cardiovascular gender differences [35]. Some other research has focused on the effects of estrogen and estrogen receptors on cardiovascular physiology and disease [36]. Isoflavone, as an analog to estrogen, may act on estrogen receptors [37] and might have potential protection to cardiovascular diseases in postclimacteric women [38]. A randomized, cross-over soy isoflavone feeding study in 12 healthy premenopausal women for about 100 days found that SI decreased estrogen synthesis [39]. However, our present study suggested that SI administrated for 4 weeks increased serum estradiol level. The discrepancy may be attributed to different treatment durations, different models, different ages, and different methods of measurement or a combination of these factors. Detailed mechanism should be investigated in further studies.

It has been proposed that ROS cause oxidative damage to a variety of cellular components and play an important role in the etiology of MI/R injury [40, 41]. Tissue injury mediated by oxygen-derived free radicals might be due to the activation of lipid peroxidation in cellular and subcellular membranes. Lipid peroxides are unstable and decompose to form a series of compounds including reactive carbonyl compounds [42]. Polyunsaturated fatty acid peroxides are able to generate MDA, which is an indicator of lipid peroxidation [43]. Previous study has reported that I/R injury could partly be prevented by a ROS scavenger [44]. After MI/R injury, MDA production was increased compared with sham group but isoflavone restored them. Our study also found that isoflavone decreased DHE fluorescence intension and expression of iNOS but increased I*κ*B*α* expression after isoflavone treatment. These findings suggested administration of isoflavone for four weeks before I/R appeared to provide obvious antioxidant effects for attenuating intracellular ROS generation in the myocardium. Accordingly, isoflavone enhanced the cardiac function after MI/R in our research, which might be beneficial to protect cardiovascular function in clinic after menopause.

Previous studies have shown conflicting results on the role of ROS on infarct size. A number of studies find that antioxidants or scavengers reduce infarct size during I/R [45, 46], while some other studies find no reduction in infarct size [47, 48]. And some reports suggested that antioxidants could only delay but not prevent manifestations of infarction [49]. These nonconsistent findings may be attributable to the difference in disease courses and drug species or doses. In our study, we found that isoflavone reduced infarct size after MI/R injury in ovariectomized rats accompanied with decreased level of oxidative stress.

On the other hand, a number of recent studies have indicated that “ischemia/reperfusion injury salvage,” including PI3K and Akt activation, played a vital role in the process of myocardium I/R [50, 51]. As is reported, one of the major targets of Akt is eNOS, which catalyzes L-arginine to produce nitric oxide (NO) and is pivotal to the cardioprotection in myocardium I/R injury [52]. In our study, we found that expression of P85*α*, phosphorylation of Akt (Ser473), and eNOS (Ser1177) decreased significantly in ovariectomized rats after MI/R injury. After isoflavone treatment, the protein expressions of PI3K/Akt/eNOS signal pathway increased, which might accelerate intracellular signaling transduction and are attributed to a marked anti-I/R injury effect. More importantly, upregulation of PI3KAkt/eNOS signal pathway induced by adequate doses of isoflavone was similar to that induced by 17*β*-estradiol supplementary. This action might be responsible for the uniform estrogen receptor, which can activate PI3K related signaling [53, 54]. It is worth noting that the attenuating effect of SI was unavailable during I/R in ovariectomized rats if LY294002 was administrated. These data suggested that soy isoflavone protects myocardial I/R injury via a PI3K dependent pathway. Meanwhile, inducible NOS (iNOS) and enhanced oxidative stress result in NO uncoupling, and loss of I*κ*B elevates inflammation. Both of them exacerbate MI/R injury [18, 55]. And isoflavone inhibited iNOS and but enhanced I*κ*B*α* expression in myocardium after I/R injury.

More intriguingly, cardiac I/R causes not only myocardial but also vascular injury [56]. It has also been proposed that reperfusion induced endothelial dysfunction, characterized by decreased endothelium-dependent vasodilatation [57]. Studies have displayed a potential role of ROS in endothelial dysfunction [58, 59]. And we found that there was serious oxidative stress in thoracic aortas after I/R injury in ovariectomized rat, which might be an important factor to impair the vascular endothelium. Chronic isoflavone treatment improved endothelium-dependent vasodilation in ovariectomized rats, which might be related to the attenuated effect on oxidative levels. Similar to the effect on myocardium, isoflavone also increased PI3K/Akt/eNOS signal pathway and inhibited iNOS but enhanced I*κ*B*α* expression in thoracic aortas, all of which are helpful to protect vascular functions.

In conclusion, although previous studies have reported beneficial effects of estrogen supplement on women's cardiovascular function, to our knowledge, our data first showed that isoflavone could evoke an enhanced improvement on myocardial I/R injury and vascular relaxation during I/R. This action was shown to be related to the strengthened action of PI3K/Akt/eNOS pathway and attenuated oxidative stress. In brief, our study might raise a possible potential therapeutic of isoflavone for postmenopausal I/R injury and even other cardiovascular diseases in clinic.

## Figures and Tables

**Table 1:** Level of body weight, uterus weight, and serum estradiol after MI/R.

	Body weight (g)	Uterus weight (g)	Serum estradiol (pg/mL)
Control	269.0 ± 11.0	0.744 ± 0.062	502.2 ± 31.5
OVS	329.8 ± 15.1^&&^	0.162 ± 0.011^&&^	194.4 ± 25.0^&&^
OVX	333.9 ± 12.3	0.157 ± 0.009	197.5 ± 23.8
CMC	328.8 ± 11.4	0.171 ± 0.018	197.3 ± 21.2
L-SI	302.6 ± 8.1	0.1953 ± 0.02684	274.8 ± 22.4
M-SI	288.6 ± 10.3^#^	0.2307 ± 0.03062	300.5 ± 21.1
H-SI	276.0 ± 8.3^##^	0.3448 ± 0.03841^#^	341.4 ± 25.1^##^
OVE	263.0 ± 7.5^##^	0.7432 ± 0.07498^##^	471.9 ± 42.5^##^

Values are mean ± SEM, *n* = 5–8. Statistical significance: ^&&^ *P* < 0.01 compared with control; ^#^ *P* < 0.05, ^##^ *P* < 0.01 compared with CMC.
